# Philosophy and Application of High-Resolution Temperature Sensors for Stratified Waters

**DOI:** 10.3390/s18103184

**Published:** 2018-09-20

**Authors:** Hans van Haren

**Affiliations:** Royal Netherlands Institute for Sea Research (NIOZ) and Utrecht University, P.O. Box 59, 1790 AB Den Burg, The Netherlands; hans.van.haren@nioz.nl

**Keywords:** high-resolution deep-ocean temperature sensors, internal waves, turbulent overturning

## Abstract

Every application may have its specifically designed sensor. For studying the effects of short-term temperature variations on life in water, a high-resolution sensor has been designed with low noise level <0.1 mK. Pro and cons of the design include its adequacy for use in large heat-capacity environments like water but less in air. The sensor can be used under high static environmental pressure of >1000 Bar (>10^8^ N m^−2^) in the deepest ocean regions. Its response time of 0.5 s in water allows quantitative studies of internal wave turbulent mixing effects, e.g., on the redistribution of matter and on nearly completely submerged human bodies. In a chain of >100 sensors, clocks are synchronized to sample within 0.02 s and a verified range of 600 m.

## 1. Introduction

Human perception is especially good in sensing relative variations. For example, 1:1000, 1 mm in a meter, length difference or obliqueness is readily seen by most but an absolute value of 1 m has a larger spread of up to 0.1 m except when estimated by the experienced carpenter. Likewise, absolute temperature T-values are considerably less precisely estimated than relative T-differences ΔT. Depending on the ambient temperature, differences of ΔT = 0.1 °C can be estimated when an arm is stimulated [1] provided the stimulus rate of change with time t is dT/dt > 0.1 °C s^−1^ [2] and both warm and cold skin receptors are functioning optimally around normal ambient skin temperature of T ≈ 32 °C [3].

Few physiological studies exist when near whole-body submersion is employed in an environment, air, and mostly fluid of varying temperatures like in a bath or in natural water-bodies such as a lake or sea. In such environments, T-differences may occur in a statically stable density stratification since warm water is less dense than cooler water in the range between 15 < T < 45 °C for which human skin T-receptors are active. In nature, vertical T-gradients are generated by diurnal solar radiation. In general, the resulting day-time temperature stratification also provides a reduced gravitational restoring force. Thus, it can support ‘internal waves’. These waves cause the stratification to vary with time and depth when measured at a fixed position in horizontal space. In order to verify the relative perception of human bodies, a series of simultaneously operating T-sensors needs to be mounted in the water body monitoring the varying stratification and perceiving the effects on partially submerged humans, as has been done near a North Sea beach [4].

The philosophy behind these internal wave measuring underwater T-sensors is the subject of the present paper. The design of these sensors has been adapted for the high-precision measurement of relative T-variations in water down to the deepest ocean trenches. The sensors bridge the gap between smooth underwater wave motions and the turbulent, sometimes violent, breaking of these waves. Using these sensors, the impact of such ‘internal-wave-induced turbulence’ on marine life is studied. Applications, recent updates, and design improvements are presented.

## 2. Sensor Design

### 2.1. Internal Wave Turbulence in the Ocean: Some Definitions

The ocean is stably stratified in density mainly because of the huge amounts of potential energy input by insolation. However, this heat input near the surface needs a mechanical source to take some of it all the way to the deep ocean bottom. The major mechanical sources are tides and atmospheric disturbances in combination with the Earth rotation [5]. Together, these sources generate less (kinetic) energy than 0.001 of the (potential) energy solar input, but their importance is significant. About a quarter of their energy goes into setting in motion the density stratification: The generation of ‘internal waves’ through interaction with underwater topography. In comparison with wind-driven surface waves that have periods O(10 s), internal waves are slow waves due to the restoring force of reduced gravity ∝ gΔρ/ρ, ρ denoting density and g is the acceleration of gravity. Their shortest ‘buoyancy’ periods are related to the density stratification varying between minutes in shallow seas and hours in the deep ocean. Their longest ‘inertial’ periods are related to the rotation of the Earth varying with latitude between half a day at the North pole and infinity at the equator. (Within latitudes ±2° from the equator, different, complex dynamics govern). Internal wave amplitudes can be over 100 m tall in the ocean, which is an order of magnitude larger than the largest surface wave amplitudes.

The loss of internal wave energy is considered the dominant deep-ocean turbulence generation process [6,7]. It is most energetic at a relatively steep sloping topography [8,9,10]. It generates about 10 times more turbulence than geothermal heat flux [11]. While the latter is mainly of the convective type of turbulence, internal wave breaking is mainly of the shear overturning type. Both types occur together during different stages of turbulence development [12,13]. For the diapycnal exchange, the turbulence type is not relevant. The mixing efficiency is relatively high for internal wave breaking in comparison with frictional flows over flat bottoms because of the rapid re-stratification by internal waves following a breaking event. 

Like in the atmosphere, ocean life except at the smallest scales would not exist without a turbulent exchange of suspended and resolved materials like sediment, nutrients, and oxygen. Most life cannot be sustained in a laminar diffusive world. Similarly, the ocean stratification and heat distribution would be quite different without turbulent exchange [5]. Like internal waves, ocean turbulent overturning varies over several orders of magnitude. The largest ‘Ozmidov’ scales of overturning in a stratified environment can be 10 to 100 m tall while the smallest ‘Kolmogorov’ dissipation scale is several mm. In time, the largest turbulent overturning occurs just shorter than the buoyancy period. Hence, it has a direct (spectral) connection with internal waves. The shortest dissipation period is about 0.01 s. This large range of scales challenges the oceanographic observer to a formidable task.

The most direct means to study the dynamics of water motions and their impact on marine life is by using pressure sensors. However, in the deep ocean, this is not trivial due to the huge static pressure to overcome amounting one atmosphere per 10 m depth increase. Second best current observations are costly and lack precision since they are either mechanical using impellor and vane or acoustical relying on sufficient scatterers that go with the flow. The third best would be the best indirect estimator of dynamical processes by the measurement of density. However, thus far, it turned out impossible to achieve sufficient precision of <0.001 kg m^−3^ by weighing water on moving platforms as well as underwater. Alternatively, one could combine the two dominant composers of density in sea water, temperature, and salt—salinity variations, but the different responses of their sensors does not allow sufficient precision. Therefore, we have to rely on the fourth best option, which is a single indirect measure of dynamics: Temperature (being the third best option in lakes).

### 2.2. Of-the-Shelf Instrumentation

In the late 1990s, standard physical oceanographic instrumentation focused on the study of the large scales with moor-able self-contained ‘Eulerian’ instruments capable of measuring for one year at a rate of once per half hour. (Two principles of measuring flow exist, which are described by and named after two 18th century mathematicians. Eulerian measurement registers flow past an instrument fixed in space. Lagrangean measurement is tracking a particle through space with time). Oceanographic of-the-shelf instruments were and are expensive and, on an ocean mooring line, they were positioned hundreds of meters apart. Their accuracy and resolution were about 0.01 °C for temperature and 0.01 m s^−1^ for current speeds. They barely resolved internal waves and not at all turbulent motions. A recent exception of point-like (1 cm^3^) sampling at a rate up to 64 Hz is Nortek’s Vector, which focuses on the small near-dissipation not the energy containing turbulence scales. As this is an acoustic instrument it relies on sufficient suspended particles scattering, which is not always the case in the deep sea.

In contrast, shipborne instrumentation lowered or tethered free-falling sends data to the connected onboard-computer at rates of up to a few 100 Hz. Under particular assumptions, such instruments resolve a number of turbulence scales but not internal waves and not the large-scale circulation. The latter could be partially monitored by repeated sampling at various locations in a quasi-synoptic hybrid Eulerian-Lagrangean fashion. Such a sampling strategy can be difficult to properly interpret dynamic processes.

### 2.3. Bridging the Gap

In order to bridge the gap between fast-sampling shipborne instrumentation and moored, near-Eulerian observations for long time-series, modern electronics are needed. Inspiration came from work by Thorpe [7] who constructed and deployed a chain of 11 T-sensors sampling 0.0001 °C resolution at a rate of once per 10 s over a range of 100 m above a 3000 m deep ocean bottom revealing highly nonlinear waves and overturns. In addition, because no affordable and adequate T-sensors were available of-the-shelf at the time, it was decided at NIOZ to start designing high-resolution T-sensors, which are the most cost-effective and feasible means to study internal waves and turbulence in the middle of the ocean spectrum. For this purpose, the aim was set to ‘10^4^’ to be a factor of 10 better in all four dimensions than equipment available at the market at the time (mid-1990s): Time rate and duration, spatial resolution and range, accuracy aims were: 10 s and 100 days, 1 m vertically and 10 m horizontally over ranges of 100 m, and 0.001 °C, respectively. Such aims of high-resolution would be adequate to bridge the gap between internal waves and the energy-containing Ozmidov-scale turbulent overturning while also monitoring some of the large-scale dynamics. The compromise did not include the resolution of the large ocean scales instead of the small Kolmogorov scales.

### 2.4. Environment

Only modern small-scale SMD-electronics are able to achieve the aims set. However, they need to be well protected from their working environment since water and especially salt water quickly and heavily deteriorated any electronic circuit-boards. O-rings and corrosion resistant (light) metals like titanium are also adequate for resisting the ambient pressure that tends to push water into the electronics container. In the deep-sea, such precautions are sufficient but, in shallow seas, one has to cope with the fouling by biomass and, near sedimentary bottoms and high currents, with the corrosion and penetration of re-suspended sediment.

## 3. Construction

Since data transfer from underwater moored instrumentation to shore is still very difficult whether acoustically via underwater buoys demanding too much power consumption, or via a cabled network being too costly and site specific, or via surface buoys because of its fishing risk and its non-taut—non-Eulerian mooring, we still rely on self-contained stand-alone sensors. This requires low power consumption design.

The heart of the underwater moorable T-sensor design is a couple of Negative Temperature Coefficient NTC-resistors that are mounted in a Wien Bridge WB-oscillator. The advantage of using semiconductors above metals (e.g., platinum) is their low noise level and relatively fast response and, thus, low power consumption. The disadvantage is their lower stability. The oscillator’s crystal has a frequency of 8 to 48 MHz depending on the sensor version. Temperature is proportional to resistance and inversely proportional to the WB-frequency. After start-up of typically 0.02 ms, stable periods of the oscillator are counted for a fixed period of time (about 0.1 s). The count variations provide an accurate estimate of rate of change of temperature. This is the same design as employed by Sea-Bird Electronics T-sensors. As several million counts cover a range of about [−10, 50] °C, the resolution is several tens of μK. The use of two resistors in one sensor improves the precision. Although suitable NTCs have a response time of τ ≈ 0.1 s or better, their protection against (salt) water considerably slows them. Because a diamond housing is too expensive and a steel one is too corrosive, we decided to use a thin laboratory glass tube: Cheap, pressure resistance and transparency for light.

The glass tip holding the NTCs is small and is about 1 mm^3^ of the size of the Kolmogorov spatial scale. However, with time constants of τ = 0.25–0.5 s, the Kolmogorov time scale is not resolved. The remainder of the electronics housing measures 0.1–0.2 m depending on the version. This is adequate to resolve the energy containing turbulence scales of up to the Ozmidov scale and in relatively shallow waters. The strength of the instrumentation is to have several tens initially and hundred(s) of T-sensors on a string currently to a sufficient range between different types and different regions of overturning.

Initially, in version NIOZ1: 32, later in version NIOZ2: 128, T-sensors were connected via cables to a central data logger and power supply unit [14,15], respectively. After considerable connection and leakage problems, it was decided to construct stand-alone self-contained sensors: Version NIOZ3 [16]. The low noise level <0.1 mK and high precision <0.5 mK NIOZ3 sensors had τ = 0.25 s in water and standard sampled at a rate of 1 Hz (adjustable) for a duration of one year on one Lithium Li C-cell battery. However, their NTCs were slightly pressure affected since they were glued to the inner wall of the glass tube. The glass tube is not vacuumed and its air maintains a laboratory pressure (~1 bar). The slight pressure effect in NIOZ3 NTCs is assumed to be caused directly by the glass instead of through air compression. It is noted that the main titanium grade-5 housing is made nearly vacuum (~0.1 bar) inside in order to keep the electronics dry and to prevent the tube from separating the sensor head during overboard operations.

**Figure 1:**
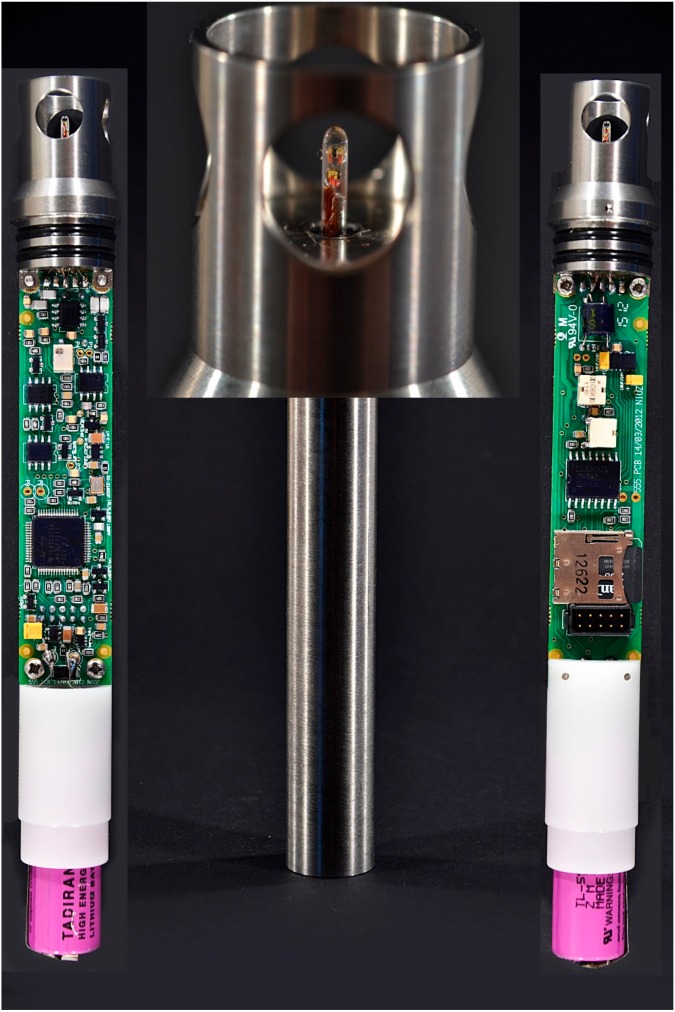
Image of NIOZ4 with micro SD-card and two induction-spools (**left**), titanium housing, sensor-tip magnification (**middle**), and main electronics (**right**). The overall length is 0.18 m and its diameter is 0.023 m and its weight is 0.23 kg.

From 2011, we developed NIOZ4, which is described here. Its principle is the same as that of NIOZ3 except the NTCs are put into a conducting paste to free them from the glass tube wall to become pressure insensitive. The time response increased to τ = 0.4–0.5 s. Power consumption was reduced by a factor of 5 partially due to the use of an 8 MHz instead of a 48 MHz WB oscillator frequency and the use of a micro-SD-card instead of a mini-SD-card for data storage with 4 kB instead of 2 kB block-sizes. NIOZ4 also contains a 3D-axis accelerator and compass to observe tilt and direction of the sensor. This additional information is rapid since it can run on 5 Hz and adds less than 5% to the power consumption of the entire sensor when sampling at the same rate as the T-sensor. This allowed for a smaller battery size. NIOZ4 runs on a single Li AA-cell battery (Figure 1). Optimized sampling strategy of 0.1 s pushes the precision into the instrumental noise of 60 μK (NIOZ3 ~45 μK) so that, with 0.3 s needed for the SD-card start-up and the writing of 500 samples at a time, steady sampling rates of 2 Hz are achievable. This would nominally drain the AA-cell in about 7 months (at 1 Hz, in >1 year) if it is as specified.

Li-batteries have a flat characteristic and a ‘sudden’ collapse upon discharge unlike exponentially deteriorating alkaline batteries. Thus, the remaining power of Li-batteries can only be established afterwards by completely draining it. This is done on a regular basis. In principle, Li-batteries have a larger capacity than alkaline batteries of the same size. They deteriorate only a few percent per year instead of 25% per year when dry-stocked and their performance drops by <10% under cold (1 to 2 °C) deep-sea temperatures at which alkaline batteries would lose more than half their power at 20 °C. These are manufacturer specifications and the latter highly depends on the amount of the current drawn. Setting NIOZ4 to measure for 0.1 s and to sample at a rate of 0.5 Hz draws about 0.12 mA of current. In practice, the T-sensor set-up drains a Li-battery twice as fast under the deep-sea when compared to near-equatorial near-surface conditions.

### 3.1. Communication and Synchronization

The tubes holding the NTCs are made of transparent glass to be able to communicate with the sensors. Via coded light-pulses emitted by an LED in the raisin cast blocking the glass-tube, the sensors can respond to requests by the operator. Requests are sent from a computer to a magnetic field that is received by one of two perpendicular spools in each sensor via induction. Examples of requests are the test of a sensor, the formatting of the data storage SD-card, the setting of deployment parameters, and the actual deployment. The latter two are given for all sensors on a string in one and the same programming. Thusfar, 515 sensors have been programmed in one session, but this number can be easily increased.

Induction is also used to synchronize the sensor clocks by sending a clock pulse from a master-clock (the ‘synchronizer’) every four hours through the conducting and strength member mooring cable. The cable is isolated from the salt water to be able to close the current loop via a brass anode and the salt water medium. In fresh water or in air, the current loop is closed via a separate return wire spiraled around the strength member cable. As a result, every sampling interval (e.g., every one second) of a profile of up to 600 m long tested so far is measured within 0.02 s. Such a snapshot over such a vertical range is not achievable using 0.7 to 1.0 m s^−1^ free-falling or shipborne lowered profilers.

### 3.2. Mounting and Deployment

Typically, 100 sensors are taped to a mooring cable of several 100 m long. Yellow, Japanese electricians tape is used, which is very sturdy under water and sticks very well to itself. This offers great flexibility and sensors have been used at intervals between 0.04 m and 2 m depending on the required configuration for study. Larger spacing than 2 m yields coarse vertical resolution. Smaller spacing than 0.2 (and 0.04) m is possible when sensors are staggered or placed horizontally.

A careful sub-surface mooring design minimizes its motions to obtain almost truly Eulerian measurements with negligible artifacts in the frequency range of interest. All buoyancy is put near the top of the mooring so that recovery after successful release of the bottom weight is smooth without entanglement of cables and potential hazards in cutting T-sensors from the cable. The taping is perfect for flexible mounting, but it is vulnerable to cable cutting/slicing. Since large-scale ocean currents are almost horizontally directed, the associated drag forces by any object obstructing the flow are nearly perpendicular to gravity (negative buoyancy direction). A balance of forces is, thus, impossible and the mooring will inevitably be deflected by a non-zero flow. The vertical deflection may never exceed half the distance between any of the instruments along the mooring line, so typically <0.5 m for a mooring holding a string of T-sensors at 1 m intervals. Hence, thin mooring cables are used of 0.005 diameter steel (0.0063 m diameter including plastic coating) that have breaking strengths of 20,000 N (2000 kg). These cables are tensioned by up to 5000 N using a weight of >500 kg and a net buoyancy of 300 kg where currents reach 0.3 to 0.4 m s^−1^. During design, the mooring motion is verified using Richard Dewey’s software (University of Victoria, Victoria, BC, Canada). During the moored period, it is always monitored by mounting at least one current meter, pressure, and tilt sensors.

The deployment of a single-line spatially one-dimensional ‘1D’ T-sensor mooring, a thermistor string, is most commonly done for free-fall oceanographic moorings. Through the aft A-frame, the top-buoy is put first in the water with the ship slowly steaming forward. The T-string is put overboard through a wide, relatively large diameter pulley or via a smoothly rounded gun-whale. Up to 100 m length of string can be put overboard manually by one or two people. In that case, the string is laid on deck in neat long loops and the mooring elements are all connected prior to the overboard operation. For the deployment of longer length strings, a 1.48 m inner diameter (1.60 m OD) 1400 pins drum is constructed to safely and fully control their overboard operation [17]. The pins guide the cables and separate them from the T-sensors in ‘lanes’ while allowing the cables to switch between lanes. The longest string deployed successfully thus far held 300 T-sensors and was 600 m long.

For the purpose of studies on the development in three dimensions of turbulent mixing by internal wave breaking above deep-ocean topography, a small-scale 3D mooring array was constructed comprising of up to 550 T-sensors [18]. The stand-alone array consists of five parallel cables 105 m long. The cables are 4 m and 5.6 m apart horizontally and are held under tension of 1000 N each by heavy buoyancy elements in a single line above. The compass/tilt information from the NIOZ4 sensors is used to monitor the stiffness of the mooring evidencing that 1000 N in each of the cables is sufficient to prevent torqueing under 0.3 m s^−1^ currents. In order to deploy five lines so close together in free fall, the entire array is folded-up into a 6 m high, 3 m diameter structure above a 750 kg weight. It is deployed in a single overboard operation, which is similar to that of a single-line mooring with the unfolding of the compacted array when hanging overboard and prior to release into free-fall (for a video see: https://www.youtube.com/watch?v=PYrK1XLbrg0&t=93s).

### 3.3. Calibration

The first 10 years of NIOZ T-sensor development, we calibrated the electronic WB-counts in situ against a shipborne Sea-Bird-911 Conductivity Temperature Depth CTD-profile. The ocean is stably stratified in temperature, but, due to the straining by internal waves and variable turbulent mixing, the stratification is spatially and temporally non-homogeneous in details. In fact, most commonly thin layers of high stratification alternate with thicker near-homogeneous layers. One can either lower the CTD-package with the moorable T-sensors mounted in the same frame to a near-homogeneous layer or one can just stop the hoisting of the CTD and wait a minute for such a layer to pass. Calibrations down to an accuracy of about 1 mK are, thus, achievable for the expected range of T-variations at the mooring site and eight to ten different temperatures. Such a calibration takes a few hours of ship-time.

**Figure 2:**
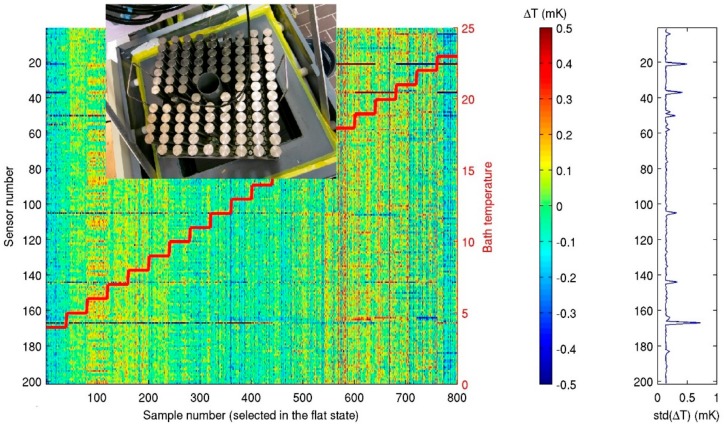
Laboratory calibration bath. Insert photo shows about 200 T-sensors upside-down in a titanium plate lifted out of the 32 L content bath. A large diagram shows the step-wise temperature in red overlaying the T-sensors discrepancy at the stable T-steps with s.d. of mean values given in the right figure. (Note about six sensors showing some anomalous behavior extending above the 0.1 mK mean level).

A calibration with a precision <0.5 mK is achievable using a thermostatic bath with constant temperature levels to within ±10^−4^ °C of their preset values (Figure 2). Such precision was obtained in a 32 L bath with some glycerine added to range between [−5, 30] °C. Up to 210 T-sensors are put with their sensor head into a 0.03 m thick titanium plate that evens out all small-scale temperature variations in the bath. Up to two high-precision SeaBird-35 Deep Ocean Standards platinum Thermometers are also put into the titanium plate for reference. Each one-degree step takes 3 to 4 hours to reach 0.1 mK stability, which is an entire calibration requiring 4 to 5 days in the laboratory.

### 3.4. Post-Processing

In spite of the large effort put into the calibration of the sensors, this does not correct for the non-randomly varying bias. For NTCs, this bias is basically due to the relatively low stability of the semiconductor resulting in electronics ‘drift,’ which is a well-known effect in temperature metrology [19]. Such a drift is noticed when temperature measurements are made in waters with T-variations in the mK-range showing horizontal bars in a time-depth image. Typical drifts are 1 mk/week initially and 1 mK/mo after aging [19,20]. However, it never disappears completely and it is temperature-dependent. During post-processing, the first step is to correct for this bias.

The correction can only be made when many (at least two but preferably >10) sensors are on a string in an environment that (eventually) is inherently stably stratified. Thus, use is made of one of the intrinsic properties of natural water bodies that are heated from above. In such an environment, vertical T-profiles averaged over at least the buoyancy period should be stably stratified because a shorter period of potentially unstable turbulent overturns are averaged out. When averaged over all internal wave scales, i.e., over the inertial period, the T-profiles should be smooth as well because the straining in step layering of short-term high and low stratification is averaged out. Any deviations from a smooth stably stratified mean profile are attributable to artificial drift, which are corrected by subtracting a constant value per sensor. In practice, the averaging period varies between the inertial period and several days depending on the natural variation of stratification locally. One can automate the above process under certain conditions [20]. This post-processing correction has the advantage that it also corrects for imperfect calibration when the T-range is not too large. Thus, a 0.1 mK precision (relative accuracy) is obtainable in certain areas.

Commonly, about 5% to 10% of the T-sensors show electronic, battery and battery-mounting, noise-tuning, or calibration problems and their data are linearly interpolated between neighboring sensors (cf. Figure 2). This affects the turbulence parameter estimates explained hereafter by less than 10%, which is within the standard error in these estimates due to the procedure of obtaining them. T-sensor retuning is done manually in the laboratory, but previous data cannot be corrected.

**Figure 3:**
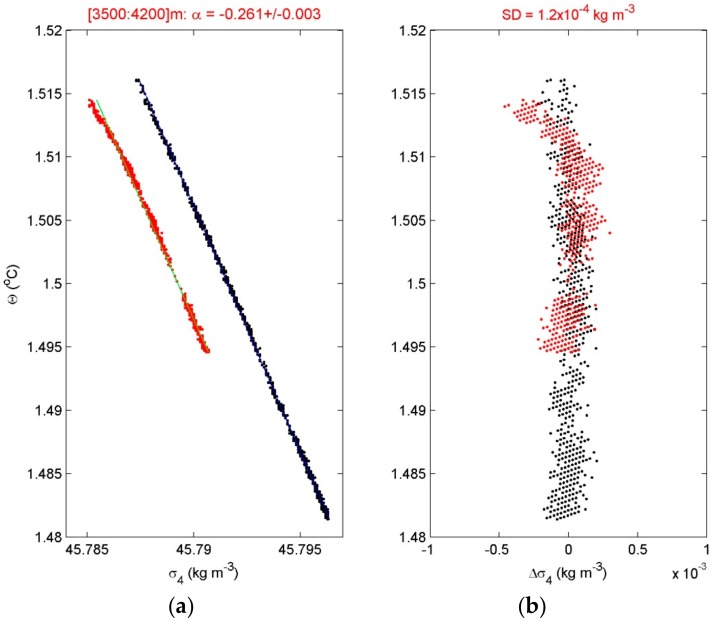
Conservative (~potential) temperature and density anomaly referenced to 4000 dbar relationship from shipborne CTD-data near abyssal hills in the near-equatorial south-Pacific DRISCOL area. (**a**) The mean slope fit is given in the left panel for CTD-observations before (red) and after (black) deployment. (**b**) Deviations from the best-fit.

The calibrated and drift-corrected data are transferred to Conservative (~potential) Temperature (Θ) values using the software described in Reference [21]. This compensates for the weak compressibility effects in water, which becomes important when T-variations are in the mK-range such as in the deep ocean. As the ocean density is also determined by salinity besides temperature, several shipborne SeaBird-911 CTD-profiles are obtained near every mooring. The CTD-data are used to establish the local temperature-density relationship for use of the moored T-sensor data as tracer for potential density anomaly variations δσ_x_, which is referenced to the pressure level x/1000 dbar of the mooring. When the linear relationship is reasonably tight (relative error of <10%), the constant gradient α = δσ_x_/δΘ indicates an apparent local thermal expansion coefficient. Such a relationship is common in relatively strong turbulence areas where internal wave breaking occurs (e.g., Figure 3) and in lakes where temperature exclusively dominates density variations with the notion that the natural thermal expansion coefficient changes the sign at about 4 °C. This relationship may not be tight in areas such as around the Mediterranean outflow of relatively warm and salty waters into a cooler and fresher Atlantic Ocean environment and in polar near-surface regions where the cold waters lead to ambient low thermal expansion coefficients with an added ice melt. In such areas, intrusions of apparent overturns may be found in T-observations that can last longer than the buoyancy period. The intrusions may qualitatively show turbulent motions, but quantitative turbulence information is no longer obtainable [22].

**Figure 4:**
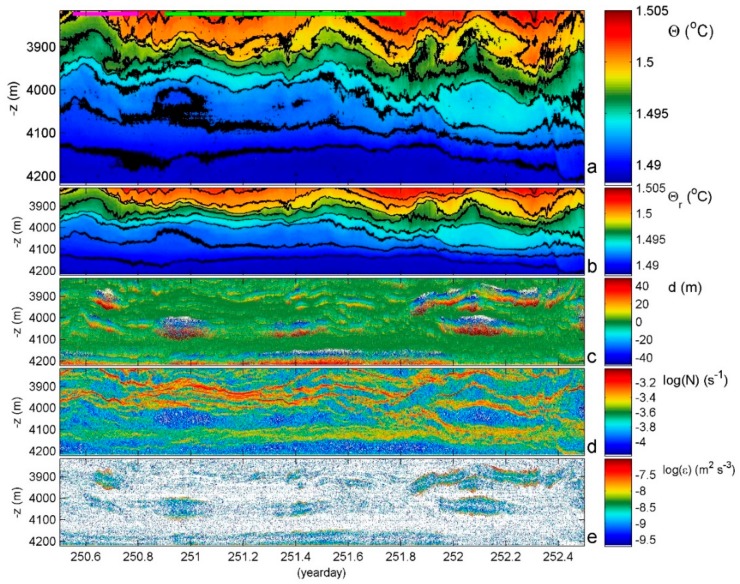
Two day—400 m time-depth T-sensor series example for a near-equatorial South-Pacific DRISCOL area (the local water depth is at the level of the horizontal axis). (**a**) Observed conservative temperature with missing sensors interpolated (see text) and black contours every 2 mK. The green and purple horizontal bars indicate the maximum and mean buoyancy periods, respectively. (**b**) Data from (**a**) reordered to stable profiles. (**c**) Displacements between data from (**a**,**b**). (**d**) Buoyancy frequency computed from data in (**b**). (**e**) Dissipation rate computed from data in (**c**,**d**).

With a tight temperature-density relationship, turbulence dissipation rate ε = c_1_^2^d^2^N^3^ and vertical eddy diffusivity K_z_ = m_1_c_1_^2^d^2^N are estimated from the T-sensor data using the reordering method of unstable points into a monotonically stable profile [23]. In this case, N is computed from the reordered profiles. Several conditions apply for the constants in the above formulas including for specifically moored T-sensors (see Appendix A). Figure 4 demonstrates the post-processing of splitting original 1 Hz profiles into reordered stable profiles and displacements. The irregular isotherm deformation in original data (Figure 4a) is completely absent in the reordered image (Figure 4b) from which the turbulent displacements (Figure 4c) are removed. The raw turbulence parameter estimates for every sensor and every (1 s) profile are averaged over depth, which is, hereafter, indicated by <…>, and time is indicated by […]. For example, [<ε>] or [<K_z_>].

## 4. Observation Examples

Observation examples are given from four different sites in this section. The locally established apparent thermal expansion coefficients varies considerably between 0.07 and 0.26 kg m^−3^ °C^−1^ and their ‘tightness’ between 0.1% and 1% of the mean value. Nonetheless, the standard errors in daily averaged turbulence parameters remain about a factor of two, as confirmed [24] by comparing moored T-sensor overturn estimates with nearby shipborne lowered ADCP/CTD data estimates using shear-scaling [25]. This error is equivalent to errors in shipborne turbulence microstructure profiler data after extensive post-processing [26]. While open ocean internal wave motions are considered as weakly turbulent [25,27], albeit their sparse breaking still provides about 100 times larger mean diapycnal exchange [<ε>] = O(10^−10^ m^2^ s^−3^), [<K_z_>] = O(10^−5^ m^2^ s^−1^) than via molecular diffusion k_d_ ≈ 10^−7^ m^2^ s^−1^, most of the internal wave breaking is now considered to be found at steep sloping topography [8,9,10]. Thereby, the importance of internal wave breaking is confirmed for mechanical mixing in the ocean at its boundaries [6,7,28]. It also confirms the rapid re-stratification or counter-gradient fluxes [29] above sloping topography rendering the internal wave induced turbulence rather effective in this case. The contrasting examples given below are from an abyssal ‘plain’ from steep slopes wave breaking and from a very shallow beach.

### 4.1. In the Deep Ocean above a Hilly Abyss

The abyssal plain is often considered as a quiescent zone where turbulent mixing is expected to be weak and because typical current amplitudes are 0.05 to 0.1 m s^−1^. Although it is known that biologically the abyssal plain may be less densely populated, it is not barren. When the food supply is locally abundant like the sinking of a whale carcass, predators come very quickly and in great numbers. This relative abundance of deep-ocean life cannot survive in a laminar world. It needs turbulence for sufficient oxygen and nutrition supplies. While the turbulence is weaker than the above sloping topography by a factor of about 100, it is a factor of 10 stronger than in the ocean interior [17]. This is seen in the example given in Figure 4 from a similar but different hilly abyss in the near-equatorial SE-Pacific (German DRISCOL area; −07°07′ S, −88°24′ W, H = 4250 m water depth, 200 T-sensors @2.0 m). Mean turbulence parameters are [<ε>] = 1.2 ± 0.8 × 10^−9^ m^2^ s^−3^, [<K_z_>] = 3.0 ± 1.5 × 10^−3^ m^2^ s^−1^).

Abyssal plains do not exist as such. There are always small ‘ripples’ or moderate ‘hills’ with which currents interact to set the ocean stratification in motion. In the 7–405 mab, meters above the bottom, two days of T-sensor image shows interfaces move by several tens of meters amplitudes, which are far exceeding the amplitudes of the largest surface waves. These internal waves are not sinusoidal ‘linear’ in appearance but deformed in triangular and other shapes after ‘nonlinear’ interaction with other internal waves and currents. The result is a wave pattern that highly varies with time including a compressing and depressing ‘straining’ of isothermal layers that greatly vary in thickness between <2 m (the present distance between the T-sensors) and 100 m (e.g., around −4000 m and day 252.1 in this example). Most of the turbulent overturning is found in or at the edges of the thicker near-homogeneous layering in which convective overturning may occur but of which the straining is governed by overall shear. The (background) shear is generally driven by the slowest internal waves near the inertial frequency, which has a four-day period. In contrast, the turbulence occurs near the buoyancy frequency like above sloping topography [29].

**Figure 5:**
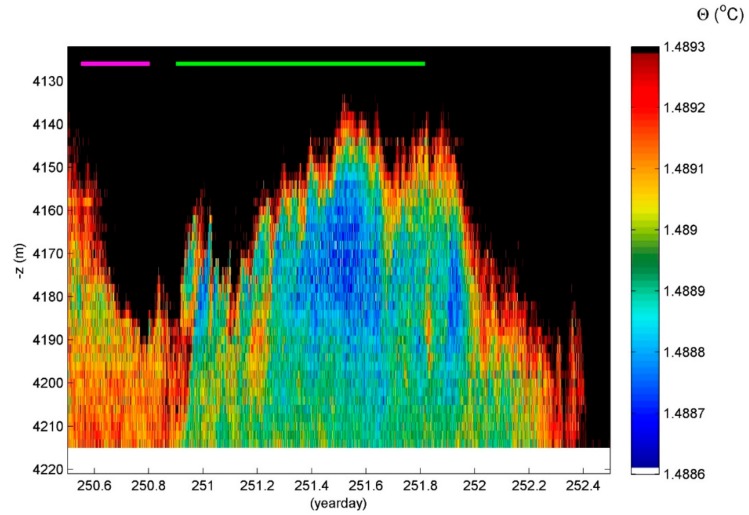
Lower 100 m above the bottom magnification from Figure 4a with a different color range of 0.7 mK overall.

At this Southern Hemisphere site, the flow over the ~100 to 200 m high surrounding hills, the nearest is 2 km away from the mooring and may set the isotherms in motion [30,31]. Yet, the temperature range is only 0.015 °C and, in the lower 65 mab, is only 0.001 °C (Figure 5), which are both well below the adiabatic lapse rate. This pushes the post-processing to great limits. The turbulent overturning is very slow and may take a day. See between days 251.1 and 252.1, which is around −4180 m. This is not related with post-processing by forcing the four-day mean to a smooth statically stable profile. It is related to the physical process of a relatively sudden upward internal wave motion, which leaves behind and below it a very weakly stratified region of about 60 m (80 m maximum) thickness. The stratification is so weak that the local buoyancy period is equal to one day, which is indicated by the green horizontal bar. This is just larger than the observed duration of the instability. Generally, turbulent overturns have the duration of up to the mean buoyancy period with thicknesses up to 100 m in the abyss (Figure 4c).

### 4.2. Over a Mid-Atlantic Ridge Crest

**Figure 6:**
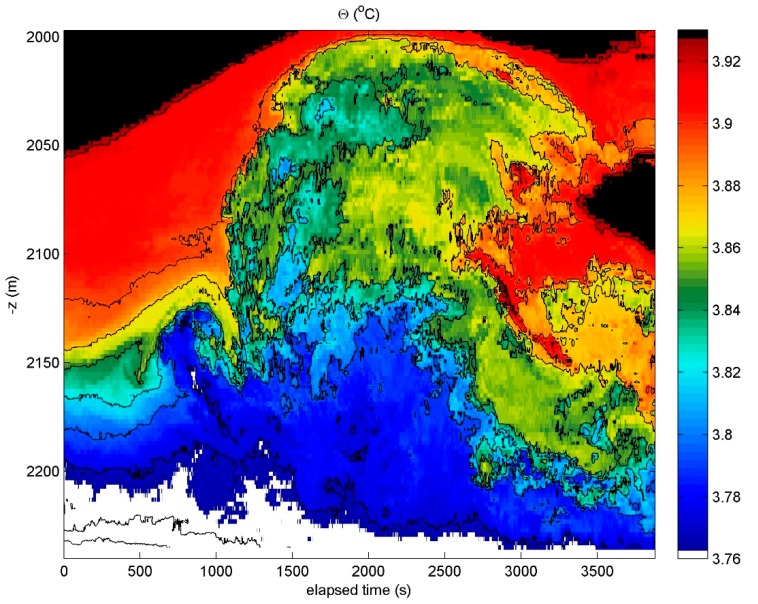
A one-hour, 200-m high upshot of a high-frequency internal wave breaking with turbulent fingers reaching out, which is measured using high-resolution T-sensors moored above a 2200 m deep slope of the Rainbow Ridge, North-Atlantic Ocean. The horizontal axis is at the sea floor. Hokusai-san [32] was right. If he could have looked into the ocean interior, the perspective would have been spot-on with the larger and smaller fingers grasping at the end of the overturning wave (here around 3000 s, 2050 m).

More intense by a factor of 10–100 is the turbulence observed over a 2250 m deep sub-ridge of the large Mid-Atlantic Ridge underwater mountain chain system using the same T-sensor array set-up of 200 sensors @2.0 m. The turbulence includes backwards breaking waves (Figure 6) that mimic sketched surface wave breaking [32]. The turbulence extends more than 200 m above the bottom [33] and its intensity is, thus, high since no trace is found of the Rainbow thermal vent system, which is only 3 km away from the mooring. These T-sensor data confirmed historic observations [34,35] that the vent is not traceable in temperature at distances >1 km away horizontally. It also confirmed the importance of turbulent mixing in canyons of underwater mountainous areas [36].

### 4.3. Over a Coral Mound

**Figure 7:**
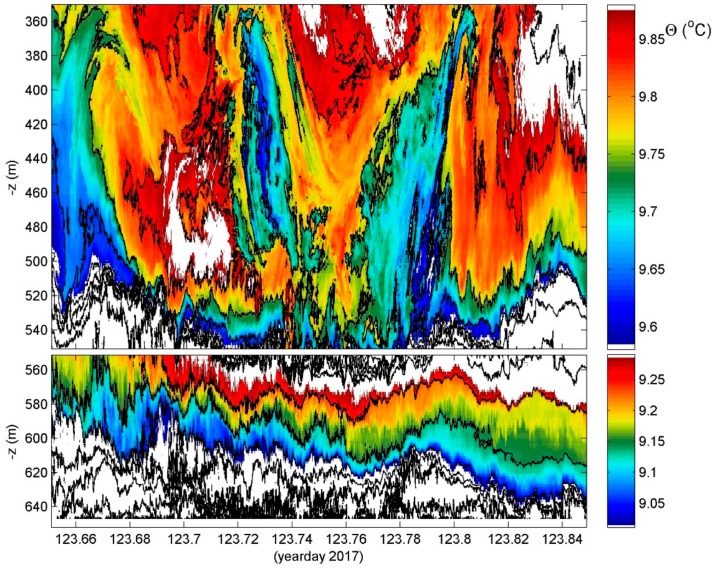
Just over 4 hours, a 300-m range example of above Haas mound off Rockall Bank, NE Atlantic (55°25′ N, −15°45′ W, H = 650 m water depth, 199 T-sensors @1.5 m). The depth-range is split in two panels with different color ranges for better visualization. In the lower panel, the horizontal axis is at the sea floor.

In further shallower waters among even more vigorous turbulence values of [<ε>] = O (10^−7^ m^2^ s^−3^), [<K_z_>] = O (10^−2^ m^2^ s^−1^) are observed above a cold-water coral mound and part of a large deep-sea bank (Figure 7). Waves near the mean buoyancy period of one hour show extremely strong shear and overturning near the sides of the mound with apparent up-shoots and down-shoots of 150 m occurring within half an hour (one tick mark in Figure 7). One can barely speak of internal wave motions as such with the image being dominated by turbulent overturning. These overturns occur during off-bank flow over the local small (150 m high) hills riddled with corals at temperatures near 10 °C. Such communities are known to thrive at such temperatures and depths where daylight barely penetrates. Yet, the strong turbulent overturning assure sufficient oxygen and nutrient supply that is not necessarily directly from the photic zone, but affecting at least (the lower) half of the water column.

### 4.4. Near Beaches

**Figure 8:**
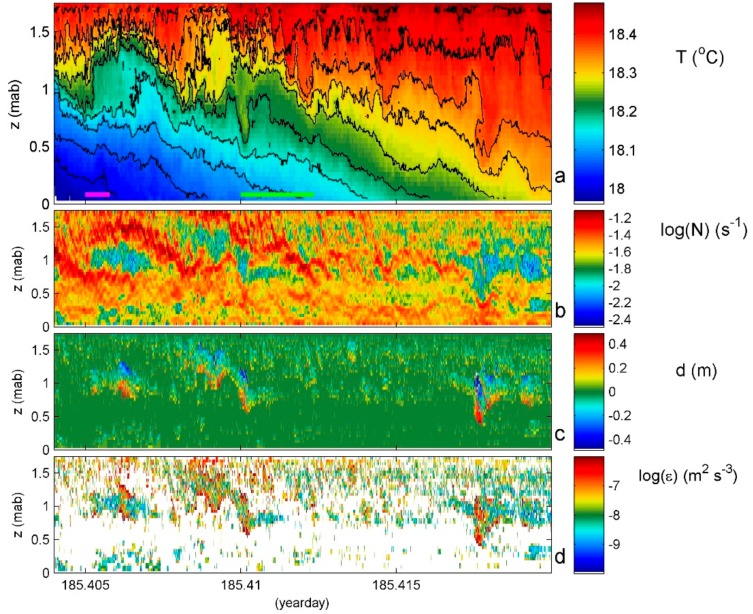
Twenty-three minutes and 1.6 m detailed example of the diurnal warming phase of the waters near an inland sea beach, ‘Ceres’ in the Wadden Sea off Texel, NL. Until day 185.41 (~10 UTC) the tide was incoming and, coincidentally, the weather was very calm (wind speeds < 3 m s^−1^; surface wave heights < 0.05 m) with overcast skies. After that, sunshine, wind speed (up to 8 m s^−1^), and surface wave height (up to 0.2 m) increased. (**a**) Observed conservative temperature with missing sensors interpolated (see text) and black contours every 0.05 °C. The green and purple horizontal bars indicate the maximum and mean buoyancy periods, respectively. (**b**) Buoyancy frequency. (**c**) Displacements between data in reordered profiles. (**d**) Dissipation rate computed from data in b and c.

A similar kind of turbulent overturning may be found at many spatial scales including those of a meter providing 10 to 100 times smaller mean values than in the previous two examples, which is observed with the same sensors but is mounted horizontally in a mast at 0.042 m distances [4]. The image Figure 8 from an inland sea beach shows the same degree of turbulent overturning as in the previous ones including, in this example, clear shear-driven Kelvin-Helmholtz instability KHi overturning at the sloping interface between incoming warmer and underlying cooler waters around 1.0 mab and days 185.406 and 185.410. The KHi have a duration of about 250 s, which is just larger than the mean buoyancy period while smaller than the local ‘minimum’ buoyancy period, with turbulent overturning cores lasting about 100 s. This is approximately equal to the shortest buoyancy period. The time-depth mean interior turbulence parameters for Figure 8 are estimated as [<ε>] ≈ 3 × 10^−8^ m^2^ s^−3^ and [<K_z_>] ≈ 8 × 10^−6^ m^2^ s^−1^. The weak diffusivity and relatively large flux (which is proportional to dissipation rate) values are associated with the large mean buoyancy frequency partially because of the stable salinity stratification adding to the temperature stratification. Secondary instabilities smaller than 0.1 m tall and 20 s in time are visible along edges of the KHi, which has also been observed in estuaries [37].

**Figure 9:**
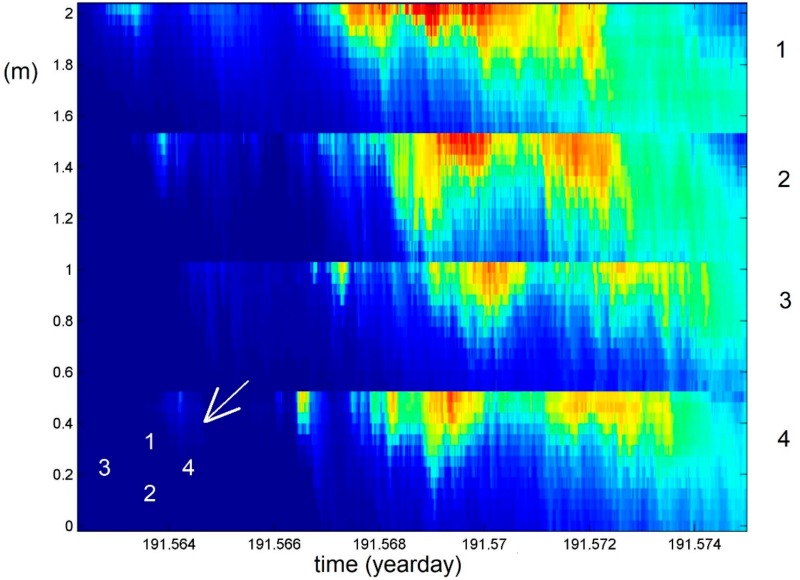
Seventeen minutes and 0.5 m depth range of slow propagation of internal wave turbulence past four mooring poles 4 m apart near Texel North Sea beach. The rippled waves came in from the NW, as indicated by the arrow. The depth range of the four poles are indicated cumulatively. The temperature range is [19.4, 19.9] °C between dark-blue and red, respectively.

Using multiple poles mounted near a North Sea beach 4 m apart horizontally, the slow propagation of about 0.03 m s^−1^ of internal waves is observed including their direction and relatively rapid deformation of the turbulent motions within a wave packet (Figure 9). This test on the de-correlation scales of internal wave-turbulence from multiple T-sensor moorings was the pre-amble for the distance between lines in the development of the foldable five-line 3D mooring [18]. The modification of turbulent overturning between the poles spaced 4 m is like watching atmospheric clouds deform and pass-by. Turbulence in the ocean seems slow, but the displaced water masses are enormous and the ocean has time.

## 5. Ongoing and Unsolved Pros and Cons

From the large bulk Reynolds number Re = 10^4^–10^6^ perspective, the ocean is turbulent in many places, which is demonstrated here with the given examples. The development of the NIOZ T-sensors has contributed to a better understanding of the internal wave turbulence transition in natural water bodies. Although one of the primary aims was to better visualize internal wave breaking when of-the-shelf instrumentation provided a too limited view, the main aim was to quantify the associated turbulent exchange. Such quantification is not possible with visually strong high-resolution acoustic imaging that can only provide qualitative results because of non-uniform shapes and various sizes of reflecting particles. Acoustics also require considerable amounts of power, so that the better instrumentation available is only shipborne and not moorable as a self-contained unit.

The pros of the developed T-sensors are their low noise level, their synchronization providing nearly true vertical profiles, and the use of many (~100) in a vertical range. This leads to the high-resolution quantification of ocean internal-wave-induced turbulence. In comparison with emerging fiber optics temperature sensors, e.g., [38], the NIOZ4 T-sensors have 1000× slower response time and 10x smaller noise level (resolution). Foremost, the need for fiber optics sensors to have both a broadband light source and a high-speed spectrometer makes them too power consumptive to be used as stand-alone systems running on a single battery in the deep-sea.

The cons of the developed T-sensors are the inevitable electronic drift and the fact that the turbulence estimates have to be made in an environment with a tight temperature-density relationship. The latter holds for all sensors lacking simultaneous salinity measurements in the ocean. The T-sensors’ sampling rate requires that the largest overturning scales contribute most to turbulence since the smaller down to Kolmogorov scales are not resolved. In addition, the severe demand on minimizing power consumption puts a constraint on the supply. In the past and more recently, the Li AA-cell batteries performed less than half as in the manufacturers’ data-sheet especially in deep-ocean cold waters. This is verified in a laboratory experiment recently (August 2018), which indicates a factor of 2 less of a power supply in 4 °C when compared to a 20 °C environment.

Progressing modern electronics may help in reducing power consumption, which will partially solve this problem. A new 12 MHz WB-oscillator requiring only 0.15 instead of 2 mA will be installed in future NIOZ6. (NIOZ5 was equipped with a Conductivity sensor besides the T-sensor, but the results were poor in determining salinity to the same degree of precision as a temperature because of matching problems with the two sensors. This sensor is now obsolete). We are rethinking the use of the C-cell Li-battery. It is planned to install thinner (0.1 mm) glass tubes providing a 10× faster response than NIOZ4. The NIOZ6 will be used in a large-scale ‘cubic hectometer’ 3D array comprising about 3000 sensors on 50 to 60 mooring lines @10 m horizontally. This array is to be deployed close to the KM3NeT cubic kilometer neutrino telescope in the deep Mediterranean off Toulon, France using their infrastructure for synchronization.

## Appendix A. Assumptions for Overturn Estimates

The first assumption is that the overturning displacements d are related to the Ozmidov scales L_O_. After suitable (rms) averaging [23], they relate to a constant ratio of c_1_ = L_O_/d_rms_ = 0.8, as empirically established as being a mean in a distribution of about one order of magnitude wide [39]. The distribution reflects the various stages of turbulence development and the two principal turbulence processes of shear-induction and convection. The high Reynolds number (10^4^–10^6^) turbulence in the stratified ocean is a mix of shear-induction and convection rendering the average ratio very close to 1, which confirms the above mean ratio. The second assumption is to use a constant value m_1_ = 0.2 for the mixing efficiency [40,41]. As mentioned above, this constant is a mean from a distribution of about one order of magnitude wide and, thus, requires some suitable vertical, horizontal, and/or time averaging. Suitable averaging is not a problem at all for rapid yoyo-ing [42] and certainly not for 1-Hz sampled moored T-sensor data, which results in turbulence estimates comparable with those from different methods to within a factor of two [24].
